# Abnormal Functional Connectivity Density in New-Onset Type 1 Diabetes Mellitus Children: A Resting-State Functional Magnetic Resonance Imaging Study

**DOI:** 10.3389/fpsyt.2020.00284

**Published:** 2020-04-17

**Authors:** Kun Liu, Jiawen Song, Jiahui Jin, Xiaoyan Huang, Xinjian Ye, Shihan Cui, Yongjin Zhou, Xiaozheng Liu, Wei Chen, Zhihan Yan, Xiaoou Shan, Yuchuan Fu

**Affiliations:** ^1^ Radiology Department, China-USA Neuroimaging Research Institute, The Second Affiliated Hospital and Yuying Children's Hospital of Wenzhou Medical University, Wenzhou, China; ^2^ Department of Pediatric Endocrine, The Second Affiliated Hospital and Yuying Children's Hospital of Wenzhou Medical University, Wenzhou, China; ^3^ Department of Psychiatry, Sir Run Run Shaw Hospital, Collaborative Innovation Center for Brain Science, Zhejiang University School of Medicine, Hangzhou, China

**Keywords:** functional connectivity density (FCD), type 1 diabetes mellitus (T1DM), new-onset, resting-state functional magnetic resonance imaging (fMRI), children

## Abstract

Type 1 diabetes mellitus (T1DM) causes cognitive changes in children, which may be due to deﬁcits in brain functions. It is unclear whether T1DM children will have brain functional changes during the initial stage of the disease. We aimed to investigate the changes in the functional brain network topology in children with new-onset T1DM. In this study, 35 new-onset T1DM children and 33 age-, sex-matched healthy controls underwent resting-state fMRI. The whole brain functional connectivity density (FCD) analysis and seed-based functional connectivity (FC) analysis were performed to investigate the changes in functional brain networks in new-onset T1DM children when compared with the controls. Pearson correlational analysis was used to explore the correlation between FCD value of differential brain areas and clinical variables in T1DM children. Compared with the controls, children with new-onset T1DM exhibited significantly decreased FCDs of the right inferior temporal gyrus (ITG) and the right posterior cingulate cortex (PCC). In the subsequent FC analysis, decreased FC was found between right PCC and right cuneus and increased FC was found between right ITG and left orbital part of inferior frontal gyrus in children with new-onset T1DM compared to the controls. The FCD values of right ITG and PCC did not correlate with HbA1c, blood glucose level before imaging, and full-scale intelligence quotient (IQ) in T1DM children. These results revealed that T1DM affect the functional activity of the immature brain at the initial stage. These findings also indicate a decrease in regional brain function and abnormalities in temporal-frontal and limbic-occipital circuitry in children with new-onset T1DM, and highlight the effects of T1DM on children's brain networks involved in visual process and memory, which may contribute to the cognition impairments observed in children with T1DM.

## Introduction

Type 1 diabetes mellitus (T1DM) is a common metabolic disease that usually occurs in children and adolescents (1). T1DM can affect the development and function maturity of the immature brain, which furtherly results in cognitive dysfunction (1). Previous studies have shown that the earlier the onset of T1DM, the higher the risk of neuropsychological deficits in later life (2, 3). Therefore, a better understanding of how T1DM affects children's brain anatomy and connectivity will help clinicians determine whether such patients are at risk of cognition decline in the future.

Magnetic resonance imaging (MRI) has been used to study the impact of T1DM on children' brain, and imaging parameters have evolved from brain structure volume to more sensitive white matter fiber integrity and brain function (4–7). Three-dimensional T1-weighted imaging (3D-T1WI) studies found that gray matter and white matter volume were reduced in several brain regions in T1DM children (4, 5). DTI studies showed that axial diffusivity was decreased in regional brain regions in T1DM children (6, 8). These findings suggest that T1DM can cause changes in gray matter and white matter structures, which may be early biomarkers of neuronal, myelin and axonal injury or degeneration. However, previous studies mainly focused on abnormal brain structural changes in T1DM, and the effect of T1DM on functional activity has rarely been studied.

Resting-state functional magnetic resonance imaging (rs-fMRI) can be used to study the functional activity of patients with T1DM (9–12). Previous rs-fMRI studies mainly focused on adult T1DM patients. Xia et al. found that decreased functional connectivity (FC) was mainly in the default mode network, which was correlated with specific impaired cognition in T1DM (12). In the study done by van Duinkerken et al., T1DM patients showed changes in FC of the subgenual cingulate cortex, mainly manifested as decreased connectivity in executive control network and increased connectivity in default mode network (13). Bolo et al. found that during hypoglycemia, the FC of the executive control network was increased in diabetic patients, which was positively correlated with HbA1c (9). FC changes in the insula and prefrontal cognitive networks may reflect adaptation to changes in brain metabolic pathways associated with chronic hyperglycemia. These studies indicated that T1DM resulted in changes in brain functional activity, and these changes were associated with cognitive impairment and blood glucose control.

As for T1DM children, the only existed fMRI study found increased FC in young T1DM children, which was positively associated with cognitive functioning (7). The results suggest that there is an abnormality in FC which is associated with the pathophysiology of cognitive impairment in children with T1DM, and that this intrinsic FC is a compensatory change in the brain for T1DM. Regardless of whether the previous fMRI studies performed in T1DM children or adults, the disease duration was more than 2 years. There is currently no fMRI study performed in new-onset T1DM children. It is still unclear how T1DM affects the FC of the developing brain at the initial stage of this disease.

Hence, the present study intends to use functional connectivity density (FCD) to investigate brain regions where there is a significant difference in FC between children with new-onset T1DM and the controls, and to correlate FCD with clinical data in T1DM children. Then, using these brain regions with FCD difference as seed points, we will analyze the FC changes between the seed points and the whole brain voxels in T1DM children. The results may reveal the pathophysiological mechanism of T1DM's impact on the developing brain.

## Methods

### Participants

From April 2016 to January 2018, 35 children with new-onset T1DM and 33 normal controls were enrolled in this study. Inclusion criteria for the T1DM group are as follows: 1) first diagnosis of diabetes in the last thirty days (14); 2) aged from 6 to 16 years; 3) complete clinical, imaging, and intelligence quotient (IQ) data; 4) right hand. T1DM was diagnosed according to the following criteria (14–16): history of polydipsia and/or polyuria, blood glucose level above 200 mg/dl, glycated hemoglobin A1C (HbA1C) above 6.5%, low insulin (fasting insulin level < 5 μIU/ml) and C-peptide level (peak C-peptide < 0.2 pmol/ml), and insulin dependency. Inclusion criteria for the control group are as follows: 1) age and gender-matched with T1DM children; 2) fasting blood glucose was lower than 6.1 mmol/L, 3) complete clinical, imaging, and IQ data; 4) right hand. Exclusion criteria for both groups are listed as follows: 1) history of preterm birth (< 37 weeks of gestation) or history of perinatal brain injury; 2) history of neuropsychiatric diseases, such as neurological or mental disorders; 3) history of using drugs which has effect on neurological function; 4) history of diseases such as nervous system infections, trauma, tumors, epilepsy, congenital malformations; 5) failure to perform MRI or MRI image quality fail to meet quality control standards. The present study was approved by the ethics committee of our hospital. Written informed consent was acquired from parents and children age 12 years and older.

### Intelligence Assessments

The general intellectual abilities of all subjects were evaluated using the Chinese Wechsler Intelligence Scale for Children or the Wechsler Intelligence Scale for Children-IV, administered by a senior medical staff who majored in intelligence assessment. Since there are many differences in the subtests of the two scales, we only use the full-scale IQ for cognitive analysis.

### MRI Scanning

Image data were acquired using a 3.0T MR750 scanner (GE, Waukesha, WI) with an eight-channel phased head coil; head movement was reduced by filling the foam padding during MR examination. All subjects should rest quietly during the examination, close the eyes, and do not think about anything. The rs-fMRI was acquired by using an echo-planar imaging sequence. The parameters are listed as follows: repetition time = 2,000 ms, echo time = 30 ms, flip angle = 90°, matrix = 64 × 64, field of view = 220 mm × 220 mm, voxel size = 3.44 mm × 3.44 mm × 4 mm, axial slices = 36, thickness/spacing = 3 mm/1 mm, and a total of 180 v were collected from each subject. A 3D T1-BRAVO sequence was used to acquire 3D high-resolution T1-weighted imaging, and the parameters are as follows: repetition time = 7.2 ms, echo time = 3.4 ms, excitation number = 1, flip angle = 12°, matrix = 256×256, field of view = 240×240 mm^2^, sagittal slices = 188, thickness/gap = 1/0 mm, voxel size = 1.0×1.0 ×1.0 mm.

### Data Preprocessing

Rs-fMRI data was preprocessed by using SPM8 (http://www.fil.ion.ucl.ac.uk/spm) and the advanced edition of Data Processing Assistant for Resting-State fMRI (DPARSFA, V4.4_180801 of DPABI_V4.0_190305, (http://www.rfmri.org ) (17–20), and the main steps include: The first 10 v of the functional images were deleted in order to exclude the influence of the initial unstable operation of the machine and the test adaptation process on the preprocessing results. Then, the remaining images were adjusted for slice timing and head movement. Subjects with a head motion of more than 3.0 mm translation or a rotation of more than 3.0° in any direction were excluded. All images were realigned and spatial normalized to the standard MNI (Montreal Neurological Institute) space. The resulting normalized images were spatially smoothed using a half-width 6 mm Gaussian kernel. All smoothed images were filtered using bandpass filtering with a low-frequency band of 0.01–0.08 Hz to reduce the effects of low-frequency drift and high-frequency noise. Linear trends were removed within each time series. Finally, sources of spurious variance such as head motion and signals from cerebrospinal fluid and white matter were regressed as nuisance signals with linear regression. Whether to remove the global signal during the post-processing of rs-fMRI data is still controversial, so we did not regress the global signal out (21–23).

### Functional Connectivity Density Mapping (Seed Definition)

After the preprocessing described above, the FCD of each voxel was calculated. FCD mapping can be derived from degree centrality (DC) calculation by calculating DC of each voxel in the whole brain. DC is an indicator of the total weights of a given node's connections and represents the degree of global connectivity of that node to the whole brain. The DC (i.e., global FCD) of each voxel in the brain networks is calculated one by one, so that a whole-brain FCD mapping can be formed. The whole brain FCD mapping is based on the binary network DC data set. In a binary network, the number of functional connections between a voxel and other voxels in the whole brain is defined as the whole brain FCD of a voxel, which is determined by calculating Pearson's correlation coefficients between a voxel and any other voxel (24). In this study, the degree centrality option tool in DPARSFA software was used to calculate the FCD value, and a threshold R of >0.25 was set to calculate the FCD (24). We also computed the FCD values using R > 0.2 and R > 0.3 to ensure that the FCD results were independent from the selection of the R thresholds. The FCD maps of the two groups were rescaled by the individual average FCD to reduce the effect of individual variability and increase the normality.

### Seed-Based Functional Connectivity Analysis

The seed-based FC analysis was performed to explore the abnormalities of FC circuits in children with T1DM. According to the FCD map, we selected the brain regions with abnormal FCD as the seeds for the whole brain FC calculation. The seed is selected by defining the functional connection density. DPARSFA was used to perform the seed-based FC analysis. For each subject, the Pearson correlation coefficients between the seed point and other voxels of the whole brain were calculated as the seed-based FC. The correlation coefficients were then subjected to Fisher z transform to improve the normality of the data, and finally the seed-based FC maps were generated.

### Statistical Analysis

The normality of demographic and clinical data was tested by Shapiro-Wilk test. Then, the demographic and clinical data were compared between the two groups using a two-sample *t*-test. The gender comparison was performed using a chi-square test. While for rs-fMRI data, we performed Lilliefors tests by using an in-house software which is based on REST (http://www.restfmri.net). Then, Voxel-wise comparisons between the two groups were performed using a two-sample *t*-test on FCD or FC maps. Finally, *P* < 0.05 (AlphaSim corrected for multiple comparisons, with a combined individual voxel *P* < 0.005 with a cluster size > 26 voxels) was set as the significance level on the resulting statistical map. Using age, gender, and head movements as covariates, we performed regression analysis to test the impact of these confounding factors on the final results. Finally, the abnormal brain regions were located by overlapping statistical T maps onto the automated anatomical landmarks (AAL) and Brodmann templates.

The brain region in which the FCD was significantly changed between the two groups was defined as the ROI, and the average FCD value of each ROI in the T1DM group was extracted, then the partial correlation analysis (age and gender as covariates) was performed between the FCD of each ROI and clinical data in SPSS 19.0. The Bonferroni corrections were used for multiple comparisons. The statistical significance level was set to *P* < 0.05.

## Results

### Demographic and Clinical Characteristics

There was a significant difference between HbA1c and blood glucose levels before imaging between the T1DM group and the control group. However, there were no significant differences in age, gender, and IQ between the two groups. Details are listed in **Table 1**.

**Table 1:** Demographic and clinical variables of two groups.

Variables	T1DM group	CON group	*P* value
N	35	33	
Age (years)	10.0 ± 2.4	9.9 ± 2.1	0.92
Sex [male/female (% male)]	16/19 (0.46)	15/18 (0.45)	0.96
HbA_1c_	13.0 ± 2.0	5.3 ± 0.3	0.00
BGL at imaging (mmol/l)	12.2 ± 4.3	4.8 ± 0.4	0.00
Hypoglycemic event	0	0	1.00
Full-scale IQ	110 ± 17	105 ± 14	0.14

T1DM, type 1 diabetes mellitus; CON, control; N, number; BGL, blood glucose level; IQ, intelligence quotient; HbA_1c_, glycosylated hemoglobin.

The data were presented as mean ± SD or median (minimal value, maximal value).

### Difference in Functional Connectivity Density Between the Two Groups

**Figure 1:**
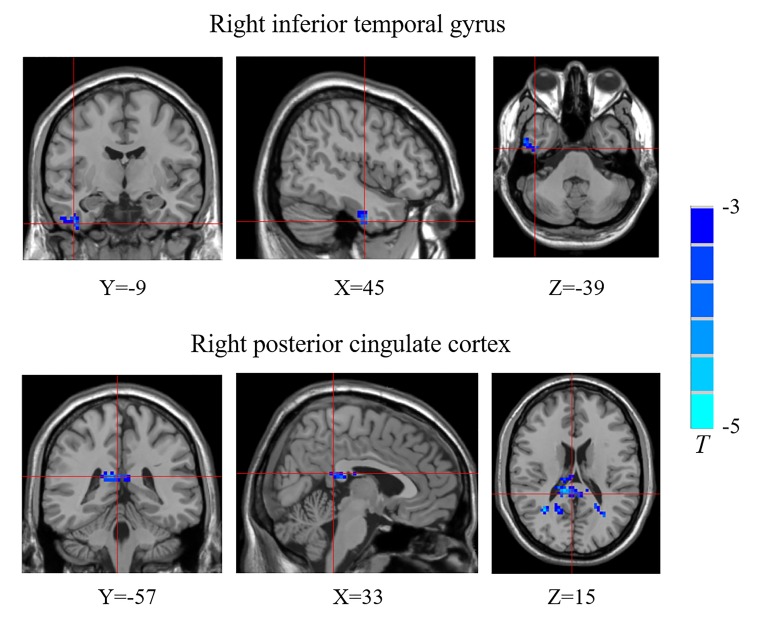
Brain regions showing decreased functional connectivity densities (FCDs) in the type 1 diabetes mellitus (T1DM) group compared with the control group. The blue color indicates that children with new-onset T1DM have lower global FCDs than the control group. The color bars indicate the T values of the contrast.

**Figure 2:**
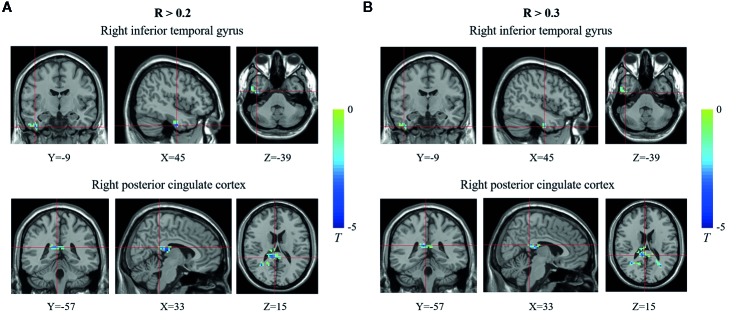
Brain regions showing decreased functional connectivity densities (FCDs) in the type 1 diabetes mellitus (T1DM) group compared with the control group using different thresholds. **(A)** R >0.2; **(B)** R >0.3. The blue color indicates that children with new-onset T1DM have lower global FCDs than the control group. The color bars indicate the T values of the contrast.

Compared with the control group, the T1DM group showed decreased FCD of the right inferior temporal gyrus (ITG) and the right posterior cingulate cortex (PCC) (**Figure 1**, **Table 2**). Validation analysis showed that the results of the FCD analysis using the thresholds of 0.2 and 0.3 were consistent with those using a threshold of 0.25 (**Figure 2**).

**Table 2:** Functional connectivity density (FCD) differences between the children with new-onset type 1 diabetes mellitus and control group.

Brain region	Brodmann area	Voxels number	MNI coordinate			T value
			*X*	*Y*	*Z*
Temporal_Inf_R	20	44	45	−9	−39	−3.747
Cingulum_Post_R	26	128	33	−57	15	−4.299

T1DM, type 1 diabetes mellitus; CON, control; MNI, Montreal Neurological Institute.

### Seed-Based Functional Connectivity Changes

**Figure 3:**
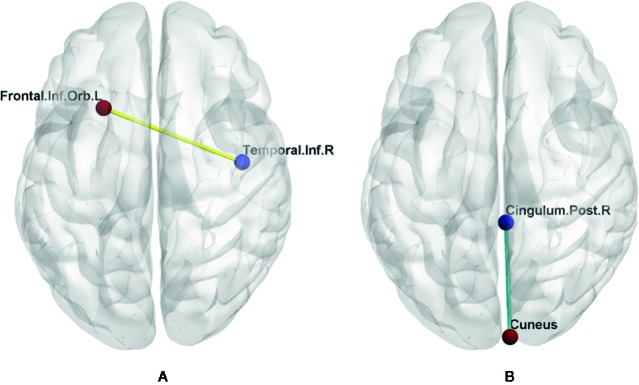
FC changes between the two groups. Left image **(A)** shows increased FC between right inferior temporal gyrus and left orbital part of inferior frontal gyrus (yellow line). Right image **(B)** shows decreased FC between right posterior cingulate cortex and right cuneus (green line). Cingulum.Post.R, right posterior cingulate cortex; Frontal.Inf.Orb.L, left orbital part of inferior frontal gyrus; Temporal.Inf.R, right inferior temporal gyrus.

There were significant seed-based FC alterations for several brain regions between the two groups when the abnormal FCD regions were selected as seed points (**Figure 3**).

When the right ITG as the seed point, the T1DM group had an increased FC value, with peak difference in the left orbital part of inferior frontal gyrus (OIFG) (**Table 3**, **Figure 2**). When the right PCC as the seed point, the T1DM group had a decreased FC value, with peak difference in the right cuneus (**Table 3**, **Figure 3**).

**Table 3:** Functional connectivity (FC) differences between the children with new-onset type 1 diabetes mellitus and control group.

Brain region	Brodmann area	Voxels number	MNI coordinate			T value
			*X*	*Y*	*Z*
Temporal_Inf_R						
Frontal_Inf_Orb_L	48	43	−24	18	−18	4.121
Cingulum_Post_R						
Cuneus_R	18	27	6	−96	15	−3.893

### Correlation Between Functional Connectivity Density Values and Clinical Variables

In the T1DM group, the right ITG and right PCC were not correlated with HbA1c, blood glucose level before imaging, and full-scale IQ (**Table 4**).

**Table 4:** Relationship between functional connectivity density (FCD) and clinical data in type 1 diabetes mellitus (T1DM) group.

Brain region	HbA_1c_		BGL at imaging		IQ		
	*r*	*P*	*r*	*P*	*r*	*P*	
Temporal_Inf_R	0.152	0.407	−0.306^a^	0.089	−0.096^a^		0.599
Cingulum_Post_R	0.157	0.389	0.195	0.284	0.147		0.423

IQ, intelligence quotient; T1DM, type 1 diabetes mellitus.

a −: decrease.

## Discussion

We aimed to investigate the differences in functional brain networks in children with new-onset T1DM. The whole brain voxel-wise FCD analysis is a data-driven method for rapidly exploring the central nodes of brain networks. Using this technique, we found that compared with the healthy control group, there was decreased FCD in the right ITG and the right PCC in children with new-onset T1DM, which were not correlated with HbA1c, blood glucose level before imaging, and full-scale IQ. The subsequent FC analysis found that compared to the normal controls, children with new-onset T1DM had increased resting-state FC between the right ITG and left OIFG and decreased FC between the right PCC and right cuneus.

Both the previous study and our study demonstrated that T1DM causes FC changes in children. FCD was showed decreased in children with new-onset T1DM in our study, indicated that T1DM can affect the functional activity of the immature brain at the beginning of the disease. However, FC was found increased in T1DM children whose disease duration over 3 years, suggested that FC was increased with the disease development (7). FC is a sign of functional reorganization in the brain. Decreased FC in our study represents a reduction in functional reorganization, which may be the potential mechanism of cognitive dysfunction observed later in life in T1DM children (25).

Specifically, we observed decreases in FCDs in the right ITG and PCC by using the whole-brain analysis. The right ITG is one part of the visual network, and is responsible for receiving visual information and processing visual stimuli (26). In a fMRI study, the PCC was activated in visual tasks, suggesting the role of PCC in the visual process (27). The decreased FCD observed for particular voxels in the right ITG and PCC in the present study, indicating that these voxels were functionally connected to fewer brain voxels and suggesting diminished function of these voxels in visual processing at the initial stage of T1DM. The cuneus also participates in visual processing (28). In the subsequent seed-based FC analysis, we found that the resting state FC between the right PCC and right cuneus in the T1DM group was significantly weaker than that in the control group. This suggested that T1DM affects the function of limbic-occipital circuitry involved in the visual network at the initial stage.

The PCC, as a part of default mode network, involves in memory and cognition (29, 30). T2DM patients with working memory impairment showed decreased FC of the PCC to widespread brain regions (31). This suggested that dysfunction of the PCC may be the neural basis of cognitive impairment. As previous reported, T1DM impaired cognitive function such as learning and memory in children (32–34). Compared with the healthy controls, T1DM patients showed significantly reduced FC between the PCC and the right middle frontal gyrus (12). In line with the previous study, our study also showed decreased FC of the PCC in children with new-onset T1DM, indicated that the functions of this brain region were diminished even at the initial stage of T1DM.

Interestingly, our study found an increase in the FC between the right ITG and the left OIFG, suggesting an abnormality in temporal-frontal circuitry. The left OIFG is related to oral and sign language grammar processing, music grammar, and language semantics, and involves in the language process (35). Except as one part of the visual network, the right ITG is also the central portion of the language formulation area region and involves in language (36–38) The increase in FC between right ITG and left OIFG may be a compensatory or adaptive response to impaired language performance in T1DM patients (39).

In addition, the present study found that in children with new-onset T1DM, the FCD of the right ITG and right PCC were not correlated with full-scale IQ. It had been demonstrated that neuronal differentiation is related to functional remodeling of neuronal networks, which will have an impact on brain FC (40). In this study, IQ was not affected by FC in the initial stage of T1DM, which may be due to the fact that our participants were newly-diagnosed and T1DM may not have enough time to influence neuronal differentiation. Another possibility is that the developing brain maintains IQ performance by compensating response to altered FC.

This study had some limitations. First, we only evaluate IQ and their relationship with FC changes in T1DM children. T1DM can cause other cognitive changes. Therefore, future research requires more cognitive tests to analyze cognitive changes and the relationship between cognition and FC. Second, due to the limited sample size, we did not assess the effect of complication (diabetic ketoacidosis) on FC. Third, this is a cross-sectional study that does not demonstrate the relationship between FC changes and the clinical features of T1DM, and therefore requires a longitudinal study.

In conclusion, this study used the FCD approach to detect brain functional abnormalities in children with new-onset T1DM, and found T1DM affect the functional activity of the immature brain at the initial stage. Our findings demonstrated decreased regional brain function and abnormal temporal-frontal and limbic-occipital circuitry at the disease's initial stage and highlighted the effects of T1DM on brain networks that mainly involve the visual process and memory, which may be the neural basis of cognitive changes in T1DM children.

## Data Availability Statement

The datasets generated for this study are available on request to the corresponding authors.

## Ethics Statement

The studies involving human participants were reviewed and approved by the ethics committee of the Second Affiliated Hospital and Yuying Children's Hospital of Wenzhou Medical University. Written informed consent to participate in this study was provided by the participants' legal guardian/next of kin.

## Author Contributions

KL and JS analyzed and interpreted the data and wrote and revised the manuscript. YF, WC, and XS analyzed the data and revised the manuscript. JJ, XH, and XY acquired the data. SC, YZ, and XL analyzed the data. KL and ZY were responsible for funding and management of the project, and revised the manuscript.

## Funding

This work was supported by the grants from Zhejiang Provincial Natural Science Foundation (LY18H070003 and LY19H180003), National Natural Science Foundation of China (81400863 and 8161101010), National Key Research & Development Program of China (2017YFC1310502), Health Department of Zhejiang province (2018KY522 and 2017ZD024).

## Conflict of Interest

The authors declare that the research was conducted in the absence of any commercial or financial relationships that could be construed as a potential conflict of interest.
